# Genome-wide identification of UDP-glycosyltransferase gene family in *Dendrobium huoshanense* and functional characterization of *DhUGT44* mediating flavonoid 7-*O*-glycosylation and cold tolerance

**DOI:** 10.3389/fpls.2026.1959202

**Published:** 2026-09-30

**Authors:** Siyuan Liu, Jiali Ren, Mengru Liu, Yongfei Yin, Tao Xu, Enhui Lai, Lanping Guo, Bangxing Han, Shanyong Yi

**Affiliations:** 1 Traditional Chinese Medicine Institute of Anhui Dabie Mountain, Anhui Engineering Research Center for Eco-Agriculture of Traditional Chinese Medicine, West Anhui University, Lu’an, China; 2 Department of Pharmacy, Anhui University of Chinese Medicine, Hefei, China; 3 State Key Laboratory for Quality Ensurance and Sustainable Use of Dao-di Herb, Institute of Chinese Materia Medica, China Academy of Chinese Medical Sciences, Beijing, China

**Keywords:** cold stress, Dendrobium huoshanense, functional characterization, genome-wide identification, UDP-glycosyltransferase

## Abstract

UDP-glycosyltransferases (UGTs) play essential roles in glycosylating plant specialized metabolites, thereby modulating their stability, solubility, and bioactivity to regulate growth, development, and environmental adaptation. *Dendrobium huoshanense*, a rare and endangered medicinal orchid endemic to China, is renowned for its rich bioactive glycosides, including polysaccharides, flavonoids, and alkaloids, which underpin its high medicinal value. However, systematic genome-wide identification and evolutionary analysis of the *UGT* gene family in *Dendrobium* remain limited, hindering their application in stress tolerance and quality improvement. In this study, we performed the first comprehensive genome-wide identification and functional characterization of the UGT family in *D. huoshanense* by integrating bioinformatics analyses with molecular experiments. A total of 71 *DhUGT* genes were identified and phylogenetically classified into 12 subfamilies. These genes are unevenly distributed across chromosomes, with tandem duplications as the primary driver of family expansion. Cis-regulatory element analysis showed that most *DhUGT* promoters are enriched with phytohormone- and abiotic stress-responsive motifs. By integrating multi-omics transcriptomic data, we identified *DhUGT44* (subfamily L) as a high priority candidate, exhibiting consistently high expression across tissues, cultivation conditions, and growth stages. Its promoter contains cold-responsive elements, and RT-qPCR confirmed significant induction under low temperature stress in leaves. Subcellular localization assays demonstrated dual localization of DhUGT44 to the plasma membrane and chloroplasts. *In vitro* enzymatic assays and transient expression in *Nicotiana benthamiana* revealed that DhUGT44 specifically catalyzes 7-*O*-glycosylation of flavonoid substrates. Furthermore, heterologous overexpression of *DhUGT44* in *Arabidopsis thaliana* significantly enhanced primary root growth under cold stress compared to wild-type plants, indicating its positive role in cold tolerance. This study provides the first systematic insight into the evolutionary dynamics and transcriptional regulation of the UGT family in *D. huoshanense*, while elucidating the flavonoid glycosylation function and cold tolerance regulatory role of *DhUGT44*. These findings offer valuable genetic resources and a theoretical basis for molecular breeding and metabolic engineering of stress tolerance and bioactive compounds in *Dendrobium*.

## 1 Introduction

Glycosyltransferases (GTs) are key enzymes that catalyze the transfer of glycosyl residues from activated nucleotide sugar donors to a wide variety of acceptor molecules (Sirirungruang et al., 2023). Based on sequence conservation, catalytic mechanisms, and substrate specificity, these enzymes have been classified into 115 families (Cantarel et al., 2009; Zhang et al., 2020). They participate in multiple essential biological processes, including polysaccharide biosynthesis, protein glycosylation, and the modification of secondary metabolites (Lund et al., 2020). Among them, the GT1 family, also known as UDP-dependent glycosyltransferases (UGTs), constitutes the most widely distributed and abundant glycosyltransferase family in plants and serves as a central player in plant glycosylation (Louveau and Osbourn, 2019; Yu et al., 2022). UGT proteins adopt a canonical GT-B fold architecture. Their *C*-terminal region contains a highly conserved 44-amino-acid plant secondary product glycosyltransferase (PSPG) motif that specifically binds UDP-sugar donors, serving as a defining hallmark of the UGT family (Wang and Hou, 2009). In contrast, the variable *N*-terminal domain determines substrate specificity and mediates the glycosylation of diverse secondary metabolites, such as flavonoids, terpenoids, and alkaloids (Bowles et al., 2005; Lu et al., 2023). Glycosylation represents one of the core regulatory mechanisms in plant secondary metabolism (Li et al., 2026). The UGT family recognizes a broad spectrum of substrates, shows tightly regulated spatiotemporal expression patterns, and can be induced by environmental stress. Together, these traits establish it as a critical functional gene family that bridges quality formation in medicinal plants and their environmental adaptability. On the one hand, UGTs directly catalyze the biosynthesis of bioactive glycosides, such as flavonoid *O*-glycosides and *C*-glycosides. Through glycosylation, these enzymes markedly improve the aqueous solubility, stability, and pharmacological activity of the substrate molecules, serving as critical regulatory nodes that determine the therapeutic efficacy of natural products in medicinal plants (Liu et al., 2024; Yu et al., 2024). On the other hand, numerous UGT members respond to a variety of biotic and abiotic stresses, including drought (Chen et al., 2024; Ma et al., 2024), low temperature (Wang et al., 2024a; Zhao et al., 2025a), and pathogen infection (Rehman et al., 2018). By modulating endogenous hormone homeostasis and promoting the accumulation of antioxidant glycosides, they enhance plant stress tolerance, underscoring their substantial potential in molecular breeding programs aimed at improving stress resilience in medicinal plants (Pei et al., 2022).

*D. huoshanense*, a rare medicinal orchid of the Orchidaceae, is officially listed in the Pharmacopoeia of the People’s Republic of China (Chinese Pharmacopoeia Commission, 2025). As a characteristic Chinese endemic medicinal resource, it has long been used in traditional medicine for invigorating the stomach, promoting fluid production, nourishing yin, and clearing heat. Modern pharmacological investigations have further demonstrated its multifaceted bioactivities, including potent antioxidant, anti-inflammatory, and immunomodulatory effects (Guo et al., 2026). In addition to the signature Dendrobium polysaccharides, flavonoid *O*-glycosides and flavonoid *C*-glycosides are characteristic metabolites that distinguish *D. huoshanense* from other *Dendrobium* species and constitute a key material basis for its core pharmacological efficacy (Yang et al., 2024; Liang et al., 2019). Collectively, these glycosides exhibit a broad spectrum of pharmacological activities, including antioxidant effects both *in vitro* and *in vivo* (Gao et al., 2022; Yuan et al., 2022), anti-inflammatory and immunomodulatory activities (Liu et al., 2025), as well as hypoglycemic (Goto et al., 2012), hepatoprotective (Park and Song, 2019), antitumor (Li et al., 2024), and neuroprotective properties (Qin et al., 2019). Current studies on *D. huoshanense* have largely focused on cultivation and propagation (Jin et al., 2016; Cai et al., 2024), polysaccharide extraction and isolation (Hu et al., 2024), and *in vitro* bioactivity evaluation of crude extracts (Zhu et al., 2023). In contrast, research on UGTs, the key enzymes responsible for glycoside modification, remains limited and fragmented. Systematic genome-wide identification, evolutionary analysis, and expression profiling of the *UGT* gene family have yet to be conducted in this species. This knowledge gap has significantly constrained advances in targeted breeding for enhanced medicinal efficacy, heterologous biosynthesis of bioactive compounds, and mechanistic understanding of geo-authentic quality formation in *D. huoshanense.*

Integrated genome-wide and transcriptome analyses represent a powerful approach to elucidate the evolutionary dynamics and functional divergence of plant gene families (Wang et al., 2024b). The strategy has been widely applied in model plants and medicinal plants, such as *A. thaliana* (Bevan et al., 2025)*, Panax ginseng* (Jiang et al., 2024)*, Coptis chinensis* (Wei et al., 2026)*, Glycyrrhiza uralensis* (Gao et al., 2024)*, and Artemisia annua* (Chang et al., 2023). It enables a bidirectional interpretation of the functional diversification patterns of gene families from both evolutionary and expression perspectives. These analyses provide a robust framework for prioritizing core candidate genes for subsequent functional validation (Xu et al., 2023). Accordingly, in this study, we systematically identified the *DhUGT* gene family in *D. huoshanense* at the genome-wide level, and dissected its evolutionary characteristics, gene structures, cis-acting regulatory elements, and multidimensional expression patterns. Combined with expression features, we screened *DhUGT44* as the core candidate gene and verified its glycosyltransferase activity and stress tolerance function via *in vivo* and *in vitro* assays. The present study aims to fill the critical knowledge gap regarding the key enzyme family involved in glycoside modification in *D. huoshanense*. It seeks to provide both a theoretical foundation and valuable genetic resources for molecular breeding of superior cultivars, biosynthetic engineering of flavonoid glycosides, and mechanistic elucidation of geo-authentic medicinal quality formation in this important medicinal plant (Shi et al., 2025).

## 2 Materials and methods

### 2.1 Identification and physicochemical characterization of the *DhUGT* gene family

The genome assembly, protein sequences, and GFF3 annotation files of *D. huoshanense* were generated in our previous study (Han et al., 2020). To identify members of the UGT family, the HMMER 3.0 software was used to perform a domain-based search against the *D. huoshanense* protein dataset with the UGT-specific Pfam model (PF00201). Hits with an E-value < 1 × 10^−10^ were retained for further analysis. The obtained candidate sequences were further subjected to conserved domain analysis using the NCBI Conserved Domain Database (CDD) (https://www.ncbi.nlm.nih.gov/cdd/), Pfam (http://pfam.xfam.org/), and SMART (http://smart.embl-heidelberg.de/). Based on multi-database cross-validation, candidate protein sequences containing a complete UGT domain and the characteristic PSPG conserved motif were retained for subsequent analyses. The physicochemical properties of DhUGT proteins, including molecular weight (MW), isoelectric point (pI), amino acid length, instability index, and aliphatic index, were predicted using the PEPSTATS tool in EMBOSS. In addition, the subcellular localization of DhUGT proteins was predicted using Plant-mPLoc (http://www.csbio.sjtu.edu.cn/bioinf/plant-multi/#).

### 2.2 Chromosomal localization and gene duplication analysis

The genomic coordinates, chromosomal physical positions, and corresponding chromosome lengths of *DhUGT* genes were retrieved from the *D. huoshanense* genome database. To elucidate the evolutionary expansion mechanism of this family, gene duplication events among *DhUGT* members were identified using MCScanX software. To achieve a comprehensive structural overview, the collinearity analysis results were integrated with chromosomal gene density profiles. Finally, TBtools software was utilized to construct the chromosomal distribution map and the intraspecific synteny diagram for the *DhUGT* genes (Chen et al., 2020).

### 2.3 Phylogenetic analysis, gene structure, and cis-acting regulatory elements

To clarify the evolutionary relationships of *DhUGT* genes, 116 full-length UGT protein sequences of *A. thaliana* were retrieved from the TAIR database (https://jbrowse2.arabidopsis.org/) following the same conserved domain screening pipeline used for *D. huoshanense*. Multiple sequence alignment of full-length UGT amino acid sequences from both *D. huoshanense* and *A. thaliana* was performed in MEGA 12. Subsequently, a molecular phylogenetic tree was constructed based on the Maximum Likelihood (ML) method, with branch support evaluated via 1000 bootstrap replicates.

To characterize the structural features of this gene family, the exon-intron organizations, including coding sequences (CDS) and untranslated regions (UTR), were parsed from the *D. huoshanense* genome GFF3 annotation file. Concurrently, conserved motifs within DhUGT proteins were identified using MEME version 5.5.0 (https://meme-suite.org/meme/) with the following parameters: the maximum number of motifs was set to 15, the motif width ranged from 6 to 50 amino acids, and all other parameters were kept at default.

For promoter analysis, the 2000-bp genomic sequence upstream of the translation initiation codon (ATG) for each *DhUGT* gene was extracted. Potential cis-acting regulatory elements were predicted using the PlantCARE database and scanned via FIMO software with a significance threshold of *P* < 1 × 10^−5^. Finally, the phylogenetic tree, conserved motifs, gene structures, and the distribution of cis-elements were integrated and visualized using TBtools (Chen et al., 2023a).

### 2.4 Transcriptomic expression profiling

Transcriptome sequencing data of *D. huoshanense* under different cultivation modes were generated using Illumina next-generation sequencing (Yi et al., 2026). Additionally, publicly available RNA-seq datasets covering various tissues and developmental stages were retrieved from the Genome Sequence Archive (GSA) of the National Genomics Data Center (NGDC, CNCB). Gene expression levels were quantified using Transcripts Per Million (TPM) values. To normalize the data, raw TPM values were log-transformed as log_2_ (TPM + 1). The normalized expression matrix was then imported into TBtools software, and bidirectional hierarchical clustering was performed using the Heatmap module to visualize the expression patterns.

### 2.5 Plant materials, cold stress treatment, and RT-qPCR analysis

Two-year-old greenhouse-grown *D. huoshanense* plants were provided by Jiuxianzun Huoshan Huoshanense Co., Ltd. (Anhui, China) and taxonomically authenticated by Professor Bangxing Han (West Anhui University). Prior to cold treatment, the plants were acclimated in a growth chamber under a 16 h light/8 h dark photoperiod with a day/night temperature of 23°C/20°C. To simulate low-temperature cold stress, the plants were exposed to 4°C, and leaf tissues were harvested at 0, 1, 3, 6, 12, and 24 h post-treatment. Three independent biological replicates were collected for each time point. All samples were immediately snap-frozen in liquid nitrogen upon collection and stored at −80°C for subsequent RNA extraction.

Total RNA from various tissues was isolated using a plant RNA isolation kit (Vazyme Biotech, Nanjing, China) and reverse-transcribed into cDNA using the HiScript II Q RT SuperMix for qPCR (Vazyme) following the manufacturer’s instructions. A genomic DNA (gDNA) removal step (37°C for 5 min) was integrated into the reverse transcription process to eliminate potential gDNA contamination and its effects on quantification accuracy. RT-qPCR assays were executed on a Roche LightCycler 480 Real-Time PCR System (Roche Diagnostics, Basel, Switzerland) using the AceQ qPCR SYBR Green Master Mix (Vazyme). The *TBP* gene was selected as the internal reference control (Yi et al., 2022). Relative gene expression levels were calculated using the 2^-ΔΔCt^ method. The experiments were conducted with three biological and three technical replicates. All primer sequences are detailed in **Supplementary Table 1**.

### 2.6 Heterologous expression and *in vitro* enzymatic assays

The full-length CDS of *DhUGT44* was amplified by PCR using specific primers designed via Primer Premier 5.0. The full-length target sequence was inserted directionally into the MBP expression vector double-digested by *Eco*R I and *Not* I, generating a recombinant construct for MBP-tagged fusion protein production. The recombinant plasmid was transformed into *Escherichia coli* DH5α competent cells, verified by colony PCR, and sequenced by Universal Biotech Co., Ltd. (Chuzhou, China). After sequence confirmation, the correct plasmid was transformed into *E. coli* BL21(DE3) cells for heterologous expression. The bacterial cells were harvested and disrupted via sonication, and the target recombinant fusion protein was purified using amylose affinity chromatography. Following affinity purification, the MBP tag was cleaved from the fusion protein using TEV protease to eliminate potential interference of the large MBP tag on UGT enzymatic activity and substrate preference. The MBP tag fragment was subsequently removed, and tag-free recombinant DhUGT44 protein was collected.

The *in vitro* glycosyltransferase activity of recombinant DhUGT44 was evaluated using UDP-glucose as the sugar donor and apigenin as the representative glycosyl acceptor. For preliminary substrate screening, flavonoid aglycones from three structural subclasses, including naringenin and daidzein, were used as glycosyl acceptors. Enzymatic products of all screened substrates were analyzed by HPLC, and semi-quantitative apparent conversion efficiencies were calculated from peak-area signals. Ultimately, apigenin was selected as the representative acceptor for subsequent detailed enzymatic characterization. The enzymatic reaction was conducted in a 100 μL reaction mixture containing 10 μL of purified protein, 0.5 mM substrate, 4 mM UDP-Glc, and 50 mM Tris-HCl buffer (pH 7.0). The reaction mixture was incubated at 40 °C for 12 h, and the reaction was subsequently fterminated by adding an equal volume of ice-cold methanol. The reaction products were analyzed using a Waters high-performance liquid chromatography (HPLC) system equipped with a Welchrom C18 column (4.6 mm × 250 mm, 5 μm) maintained at 30 °C. The mobile phase consisted of solvent A (0.1% formic acid in water, v/v) and solvent B (acetonitrile) at a flow rate of 0.8 mL/min, Detailed conditions are shown in **Supplementary Tables 2**, **3**. Liquid chromatography-tandem mass spectrometry (LC-MS) analysis was executed on a Q-TOF 5600+ mass spectrometer equipped with a Turbo V ion source (AB Sciex, Framingham, MA, USA), employing the same chromatographic and analytical conditions as described for the HPLC analysis. For structural confirmation of the glycosylated product, nuclear magnetic resonance (NMR) spectroscopy was carried out. The purified product was dissolved in DMSO-d_6_, and ¹H-NMR and ^13^C-NMR spectra were acquired on a Bruker AVANCE III 600 MHz spectrometer at room temperature. Chemical shifts were referenced to residual solvent peaks. The NMR data were compared with published literature to determine the glycosylation position and chemical structure of the enzymatic product.

### 2.7 Enzymatic properties

Enzymatic activity assays of the recombinant DhUGT44 protein were performed using apigenin as the acceptor substrate and UDP-Glc as the sugar donor, and the effects of pH, temperature, reaction time and divalent metal ions on its catalytic activity were systematically evaluated. For pH optimization, enzyme activity was determined using two 50 mM buffer systems: Na_2_HPO_4_-NaH_2_PO_4_ phosphate buffer (pH 6.0-8.0) and Tris-HCl buffer (pH 7.0-9.0). The optimal reaction temperature was assessed in 50 mM Tris-HCl buffer (pH 7.0) following incubation at 4, 30, 40, 42, 50, 60 and 70 °C for 2 h. For the time-course assay, reactions were carried out in 50 mM Tris-HCl buffer (pH 7.0) at 40 °C with 0.5 mM apigenin as the substrate, with aliquots collected at 30 min, 1 h, 2 h, 6 h, 12 h, 16 h and 24 h. The impacts of divalent metal cations (Mg^2+^, Ca^2+^, Zn^2+^, Co^2+^, Ba^2+^ and Mn^2+^) and EDTA were evaluated by supplementing each reagent at a final concentration of 10 mM into the reaction system buffered with 50 mM Tris-HCl (pH 7.0), followed by incubation at 40 °C for 1 h. All reactions were immediately terminated by the addition of two volumes of pre-chilled methanol. After centrifugation at 14000 rpm and 4 °C for 4 min, the supernatants were filtered and analyzed by HPLC. All assays were conducted in triplicate, and substrate conversion rates were calculated from the corresponding HPLC peak areas.

### 2.8 Subcellular localization and transient expression analysis in *N. benthamiana*

The full-length coding sequence of *DhUGT44* without the stop codon was directionally inserted into the pCAMBIA1302-GFP vector to construct the fluorescence fusion expression vector 35S::*DhUGT44*-GFP driven by the CaMV 35S promoter. After sequencing verification confirmed that the recombinant vector contained no mutations and maintained the correct reading frame, it was introduced into competent cells of *Agrobacterium tumefaciens* strain GV3101 via electrotransformation. PCR-confirmed positive single colonies were selected and inoculated into 10 mL of liquid YEP medium supplemented with appropriate antibiotics, followed by shaking incubation at 170 rpm for 12 h. Agrobacterium cells were collected by centrifugation at 4000 rpm for 4 min, and the supernatant was discarded. The cell pellet was resuspended in an infiltration buffer (10 mM MgCl_2_, 10 mM MES, 100 μM acetosyringone), and the optical density OD_600_ of the bacterial suspension was adjusted to approximately 0.6. Healthy and undamaged *N. benthamiana* plants exhibiting uniform growth were selected; the bacterial suspension was slowly infiltrated into the abaxial epidermis of the leaves using a needleless 1 mL syringe, and the treated leaves were clearly marked.

The infiltrated tobacco plants were allocated into two experimental groups for subsequent treatments. The first group was incubated under continuous darkness for 48 h, after which the GFP signals were visualized using laser scanning confocal microscope (LSCM) to characterize the subcellular localization of the DhUGT44 protein. The second group was initially kept in the dark for 24 h and then transferred to a standard photoperiod (16 h light/8 h dark; light intensity: 60-100 μmol·m^−2^·s^−1^) for an additional 2 days. Subsequently, the substrate apigenin (**1**) and UDP-Glc were co-injected into the previously infiltrated regions. Following another 2 days of cultivation, the tobacco leaf samples were harvested. The collected samples were lyophilized, extracted with methanol, and filtered through an organic membrane filter. LC-MS was subsequently employed to identify the catalytic products, thereby validating the *in vivo* enzymatic function of DhUGT44.

### 2.9 Genetic transformation and stress tolerance phenotypic identification of *A. thaliana*

Owing to the immature and inefficient stable genetic transformation system of *D. huoshanense*, substantial technical obstacles hinder the generation of transgenic lines and in planta functional characterization of target genes. Thus, *A. thaliana* was utilized as a heterologous expression system to dissect the biological function of *DhUGT44* in planta. The full-length CDS of *DhUGT44* was codon-optimized for *A. thaliana* and synthesized. This target fragment was cloned into the pNC-Cam1304-35S vector under the control of the cauliflower mosaic virus (CaMV) 35S promoter. After sequencing verification, the recombinant plasmid was introduced into *Agrobacterium tumefaciens* strain EHA105 using the heat-shock method. The Agrobacterium culture was subsequently propagated in liquid YEP medium supplemented with appropriate antibiotics until OD_600_ reached 0.8-1.0. Cells were harvested by centrifugation and resuspended in a floral dip infiltration solution containing 5% (w/v) sucrose and 0.05% (v/v) Silwet L-77 to adjust the final OD_600_ to approximately 0.8. Wild-type *A. thaliana* (Columbia-0, Col-0) plants were transformed using the floral dip method. Harvested T1 seeds were surface-sterilized and sown on half-strength Murashige and Skoog (1/2 MS) solid medium supplemented with 25 mg/L hygromycin for resistance screening (**Supplementary Figure 1**). Transgenic lines were selected based on the resistance segregation ratios in the T2 generation, and three independent homozygous T3 overexpression (OE) lines were ultimately obtained for subsequent stress tolerance assays. *Arabidopsis* plants were cultivated in a growth chamber maintained at 23°C under a 16 h light/8 h dark photoperiod.

Seeds of wild-type (Col-0) and homozygous *DhUGT44*-OE lines were surface-sterilized and sown on antibiotic-free 1/2 MS solid medium, then stratified at 4 °C in the dark for 3 days to synchronize germination. After stratification, plates were placed vertically and incubated at 23 °C under a 16 h light/8 h dark photoperiod until seedlings reached a primary root length of approximately 1 cm. Seedlings with uniform growth were randomly divided into control and cold stress groups. The cold stress group was incubated at 4 °C under the same photoperiod for 3 days, while the control group was maintained under standard growth conditions at 23 °C. After cold treatment, all seedlings were transferred back to standard growth conditions and cultivated for another 3 days, and then primary root length was measured. At least 30 seedlings per genotype were used for each biological replicate. Data are presented as mean ± standard error, and statistical significance was determined using one-way ANOVA followed by Tukey’s multiple comparison test.

## 3 Results

### 3.1 Identification and physicochemical characterization of the *UGT* gene family in *D. huoshanense*

A total of 71 UGT family members were identified in *D. huoshanense* through comprehensive bioinformatics analyses and were systematically designated *DhUGT1*-*DhUGT71*. The detailed annotation information for all identified genes is provided in **Supplementary Table 4**. The physicochemical characterization revealed considerable variation among DhUGT proteins. Protein lengths ranged from 179 to 1094 amino acids (aa), with DhUGT33 encoding the longest protein (1094 aa) and DhUGT26 the shortest (179 aa). The molecular weights of the corresponding proteins ranged from 20.0 to 124.0 kDa, with an average of 54.98 kDa. The theoretical pI ranged from 4.57 to 8.15, with an average value of 6.10. Among the 71 DhUGT proteins, 35 members (49.30%) exhibited pI values above the family average, whereas the overall distribution suggested that most DhUGT proteins are weakly acidic. Analysis of protein stability and Grand average of hydropathicity showed that the aliphatic index ranged from 79.94 to 108.08, with an average of 92.37. The average instability index was 46.10, and only 10 proteins (14.1%) had instability indices below 40, the threshold for stable proteins, indicating that the majority of DhUGT proteins are predicted to be unstable. Furthermore, the grand average of hydropathicity (GRAVY) values were below zero for 43 DhUGT proteins (60.56%), suggesting that most family members are hydrophilic. Subcellular localization prediction revealed that DhUGT proteins were mainly distributed in chloroplasts and cytoplasm, followed by the plasma membrane. Only a small number of family members were predicted to localize to mitochondria or the nucleus.

### 3.2 Chromosomal localization and gene duplication event analysis

Intraspecific synteny analysis identified three pairs of segmentally duplicated homologous genes within syntenic blocks, including *DhUGT27*/*DhUGT45*, *DhUGT28*/*DhUGT70*, and *DhUGT29*/*DhUGT45* (**Figure 1A**). Chromosomal localization analysis showed that the 71 *DhUGT* genes were unevenly distributed across 15 chromosomes and one unanchored scaffold (Scaffold1907) in *D. huoshanense* (**Figure 1B**). Chromosome 6 contained the largest number of *DhUGT* genes (11 genes, 15.5%), followed by chromosomes 2 and 13 with 8 (11.3%) and 7 (9.9%) genes, respectively. Chromosomes 10 and 16 each harbored 6 *DhUGT* genes, whereas chromosomes 3, 4, 14, and 18 contained only 2 genes each (2.8%). The majority of the genes arose via tandem duplication. The detailed chromosomal locations and gene duplication types are presented in **Supplementary Table 5**.

**Figure 1:**
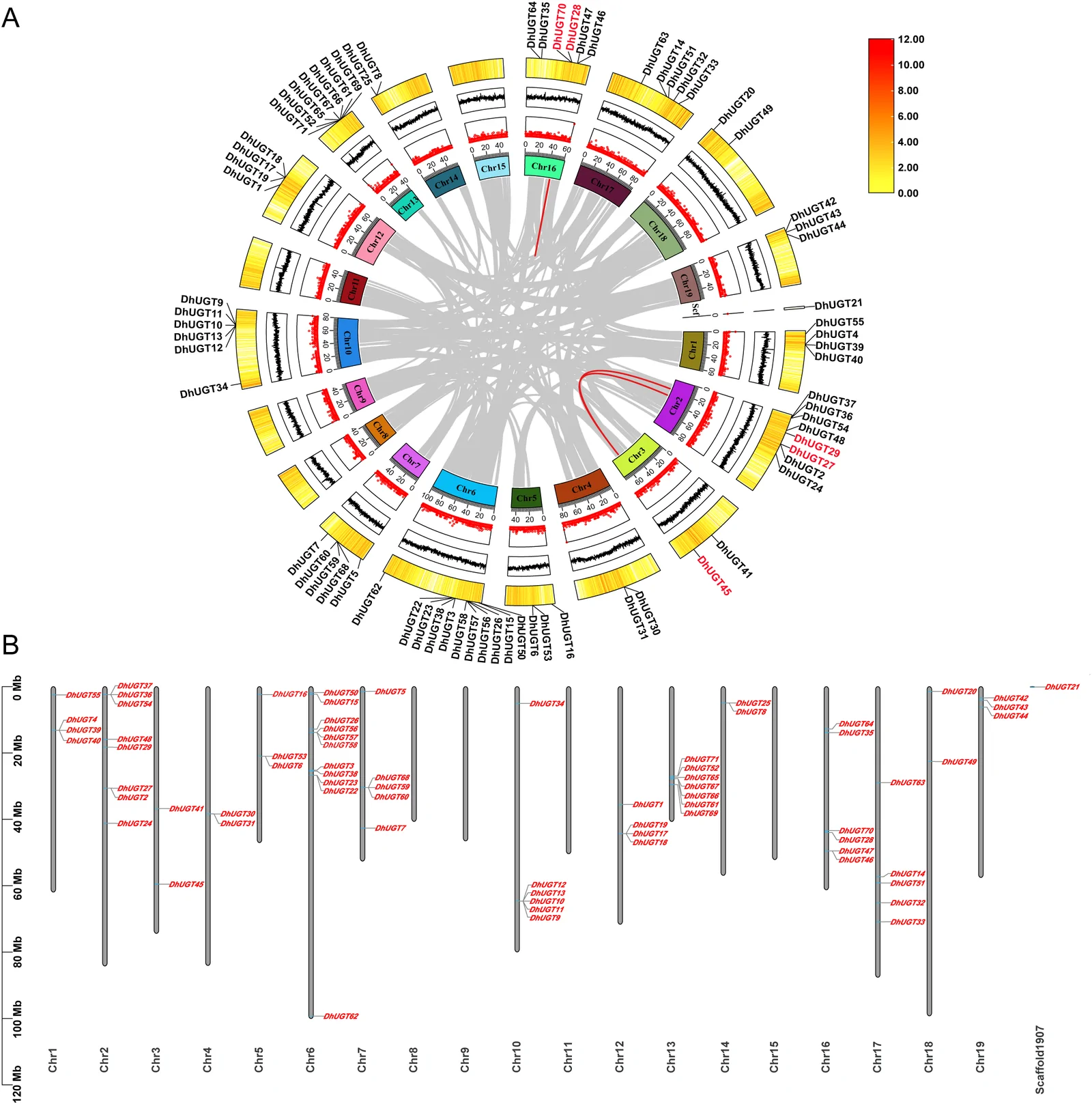
Chromosomal distribution and gene duplication events of *DhUGT* genes. **(A)** Intraspecific synteny analysis of *DhUGT* genes. Chromosomes are depicted as colored blocks, with individual gene names labeled around the periphery. **(B)** Chromosomal mapping of *DhUGT* genes. Gene names and chromosome numbers are highlighted in red and gray. The scale bar on the left indicates the physical length of the chromosomes. The precise genomic positions of *DhUGT* genes are annotated on the right side of each chromosome.

### 3.3 Phylogenetic analysis of the DhUGT family

To elucidate the evolutionary characteristics and taxonomic status of the UDP-glycosyltransferase gene family in *D. huoshanense*, a phylogenetic tree was constructed based on the multiple sequence alignment of 71 UGT protein sequences from *D. huoshanense* and 116 from the model plant *A. thaliana*. The results demonstrated that all members were clustered into 12 major evolutionary groups, designated as groups A-L (**Figure 2**). The distribution of DhUGT members across these groups exhibited prominent unevenness. Group E was the largest, comprising 13 DhUGT members (18.3%), followed by group D with 11 members (15.5%). In contrast, groups K and F were the least represented, each containing only a single DhUGT member.

**Figure 2:**
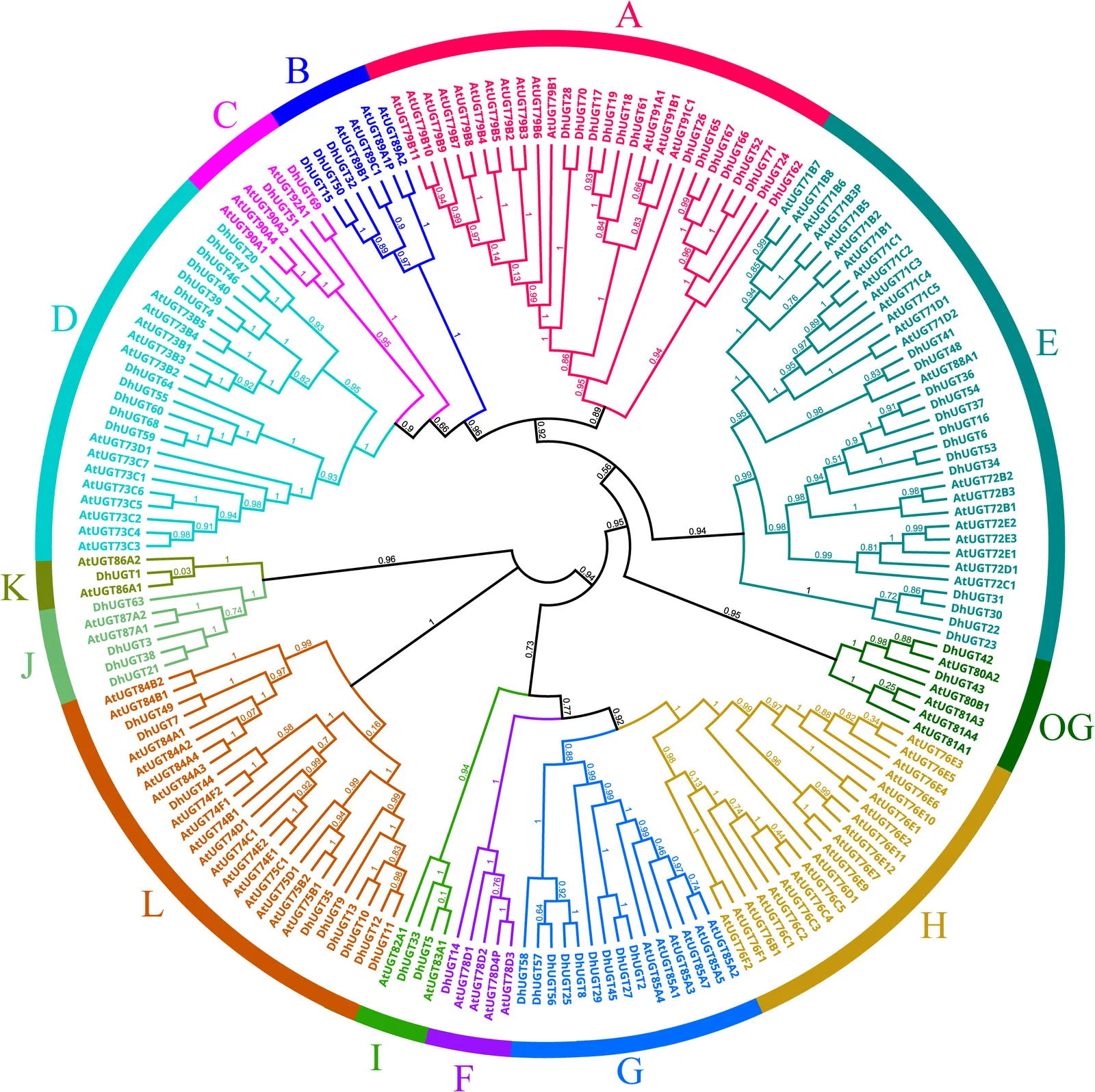
Phylogenetic analysis of UGT family proteins from *D. huoshanense* and *A. thaliana.* The phylogenetic tree was constructed using the Maximum Likelihood (ML) method in MEGA 12 based on full-length protein sequence alignment. The tree comprises UGT members from *D. huoshanense* and *A. thaliana*. Distinct colored sectors designate different groups **(A–L)**, with “OG” representing the outgroup. Capital letters on the outer ring label the respective evolutionary clades. Numbers at nodes indicate the bootstrap support values based on 1000 replicates.

### 3.4 Conserved motifs and gene structure

To further investigate the sequence conservation and evolutionary characteristics of gene structures in the *DhUGT* genes, a phylogenetic tree was constructed based on the full-length amino acid sequences of the 71 DhUGT proteins, followed by integrated analyses of conserved motifs and gene structures (**Figure 3**). The subgroup classification inferred from the phylogenetic tree was highly consistent with the distribution patterns of conserved motifs, supporting the reliability of the phylogenetic grouping (**Figure 3A**).

**Figure 3:**
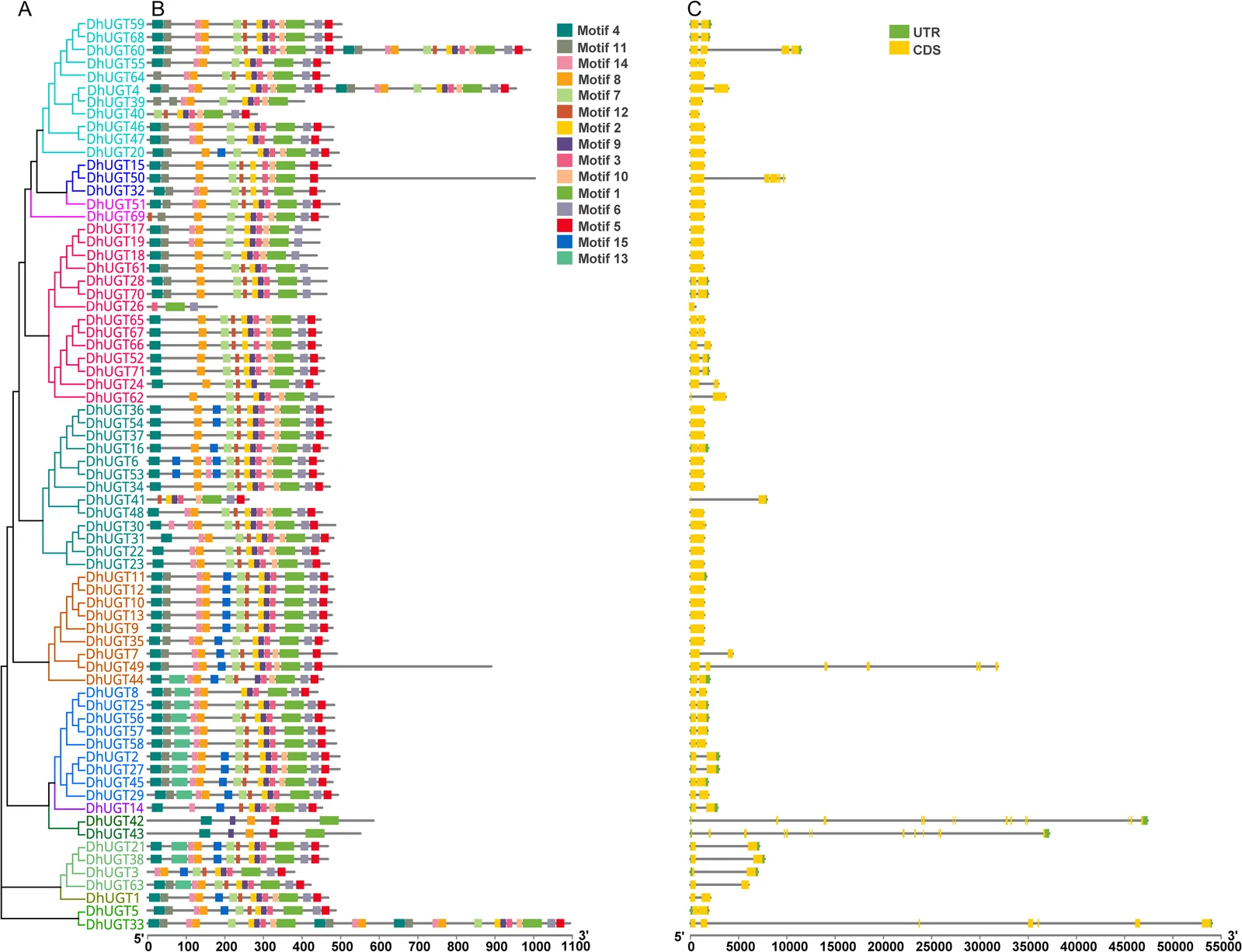
Sequence characteristics of the *DhUGT* gene family. **(A)** Phylogenetic tree of the *DhUGT* gene family. **(B)** Conserved motifs identified in DhUGT proteins. **(C)** Gene structures of *DhUGT* genes. Green boxes represent untranslated regions (UTRs), yellow boxes represent coding sequences, and gray lines indicate introns.

Conserved motifs in the DhUGT proteins were identified using the MEME program (**Figure 3B**). A total of 15 conserved motifs, designated Motif 1-15, were identified, and the sequence logos of these motifs are presented in **Supplementary Figure 2**. Motif 1 was present in all DhUGT proteins and corresponded to the highly conserved PSPG motif, which is characteristic of the UGT family. Motifs 2, 3, 7 and 8 were also widely distributed and highly conserved among most DhUGT proteins. Specifically, Motif 2 and Motif 3 were detected in 68 and 66 proteins, respectively, whereas Motifs 7 and 8 were each identified in 65 members. Overall, the composition and arrangement of conserved motifs exhibited pronounced subgroup-specific patterns, with members within the same subgroup displaying highly similar motif organization. For example, Motif 13 was exclusively detected in members of subgroups G and J. In contrast, several DhUGT proteins contained relatively few conserved motifs, suggesting sequence divergence. DhUGT26 contained only three motifs (Motifs 1, 3 and 6), whereas DhUGT42 and DhUGT43 each possessed only five motifs (Motifs 1, 4, 5, 8 and 9).

Gene structure analysis revealed substantial structural diversity within the DhUGT family during evolution (**Figure 3C**). The number of exons ranged from 1 to 14, and more than 60% of the *DhUGT* genes exhibited compact gene structures containing only one or two exons, a characteristic feature commonly observed in plant *UGT* gene families. In contrast, only a few genes contained a relatively large number of exons. Among them, *DhUGT5* possessed 14 exons, representing the highest exon number in the family.

### 3.5 Analysis of cis-acting regulatory elements and transcription factor binding sites

To elucidate the transcriptional regulatory basis of the *DhUGT* gene family, the 2-kb promoter sequences upstream of the translation initiation site (ATG) of the 71 family members were extracted and subjected to cis-acting regulatory element annotation and transcription factor binding site (TFBS) prediction. The statistical results of the cis-acting elements are summarized in **Supplementary Table 6** and **Supplementary Figure 3**, and the distribution profiles of the TFBSs are presented in **Supplementary Figure 4**.

Cis-acting element annotation revealed a total of 20 functionally defined categories of cis-elements within the promoters of *DhUGT* genes, spanning four major regulatory classes: light, phytohormone, abiotic stress, and tissue-specific responses. Light-responsive elements were the most widely distributed type, occurring in the promoters of all *DhUGT* genes, with the highest copy numbers observed in *DhUGT32* (48 copies), *DhUGT9* (26 copies), and *DhUGT26* (23 copies). Among the hormone-responsive elements, ethylene-, methyl jasmonate (MeJA)-, and abscisic acid (ABA)-responsive elements were the most prevalent. The highest copy numbers for these elements were identified in *DhUGT30* (11 copies for ethylene), *DhUGT24* (14 copies for MeJA), and *DhUGT55* and *DhUGT44* (13 copies each for ABA). Gibberellin- and auxin-responsive elements were also detected across various promoters, albeit with lower overall abundance. Regarding abiotic stress-related elements, low-temperature-responsive elements were identified in 55 *DhUGT* genes, peaking in *DhUGT65* (14 copies) and *DhUGT56* (13 copies). Wound-responsive elements were enriched in *DhUGT56* (8 copies), whereas drought-responsive elements were predominantly located in the promoters of *DhUGT65* and *DhUGT51*. The highest copy number of general stress-responsive elements was recorded in *DhUGT26* (8 copies). Among tissue-specific elements, meristem-specific elements were most abundant in *DhUGT61* (6 copies) and *DhUGT1* (5 copies), while sulfur-responsive elements each possessed 5 copies in *DhUGT21* and *DhUGT29*.

TFBS prediction identified more than 2500 potential binding sites within the promoter regions of the 71 *DhUGT* genes, belonging to 50 distinct transcription factor (TF) families. Notably, the BBR-BPC family binding sites occurred with the highest frequency, widely distributed across the vast majority of *DhUGT* promoters and forming dense site clusters in the promoter regions of genes such as *DhUGT2*, *DhUGT6*, and *DhUGT29*. The Dof and ERF families ranked second in abundance, exhibiting tandem repeat distributions in multiple gene promoters. Furthermore, binding sites for the B3, AP2, MIKC_MADS, and GRAS families were also highly prevalent and frequently located adjacent to Dof or BBR-BPC sites. Distinct *DhUGT* genes displayed pronounced heterogeneity in their TFBS composition: the *DhUGT1* promoter was enriched with sites from the G2-like, Dof, GATA, and BBR-BPC families; *DhUGT5* and *DhUGT57* were dominated by Dof and B3 family sites; and the *DhUGT41* promoter featured the highest proportion of ERF family sites alongside multiple LBD family binding sites. Stress-responsive TF family sites, including NAC, WRKY, bZIP, and bHLH, were detected across various genes, though their overall distribution remained relatively scattered.

### 3.6 Expression profiles of *DhUGT* genes across diverse tissues, cultivation modes, and developmental stages

To systematically dissect the transcriptional expression patterns of the *DhUGT* gene family, deep mining was performed on transcriptome datasets encompassing diverse tissues, cultivation modes, and developmental stages. Hierarchical clustering heatmaps were utilized to present the spatiotemporal expression and environmental response characteristics of these genes. Analysis of the tissue-specific transcriptomic profiles (**Figure 4A**) revealed distinct tissue expression specificity among DhUGT family members. Specifically, *DhUGT1* was identified as a stem-specific, highly expressed gene. *DhUGT13*, *DhUGT17*, and *DhUGT62* displayed pronounced root-preferred expressions, whereas *DhUGT52*, *DhUGT64*, *DhUGT19*, and *DhUGT40* exhibited significant transcript enrichment in leaves. Moreover, *DhUGT44*, *DhUGT34*, and *DhUGT21* maintained consistently high transcript levels across all examined tissues (roots, stems, and leaves), exhibiting typical constitutive expression characteristics.

**Figure 4:**
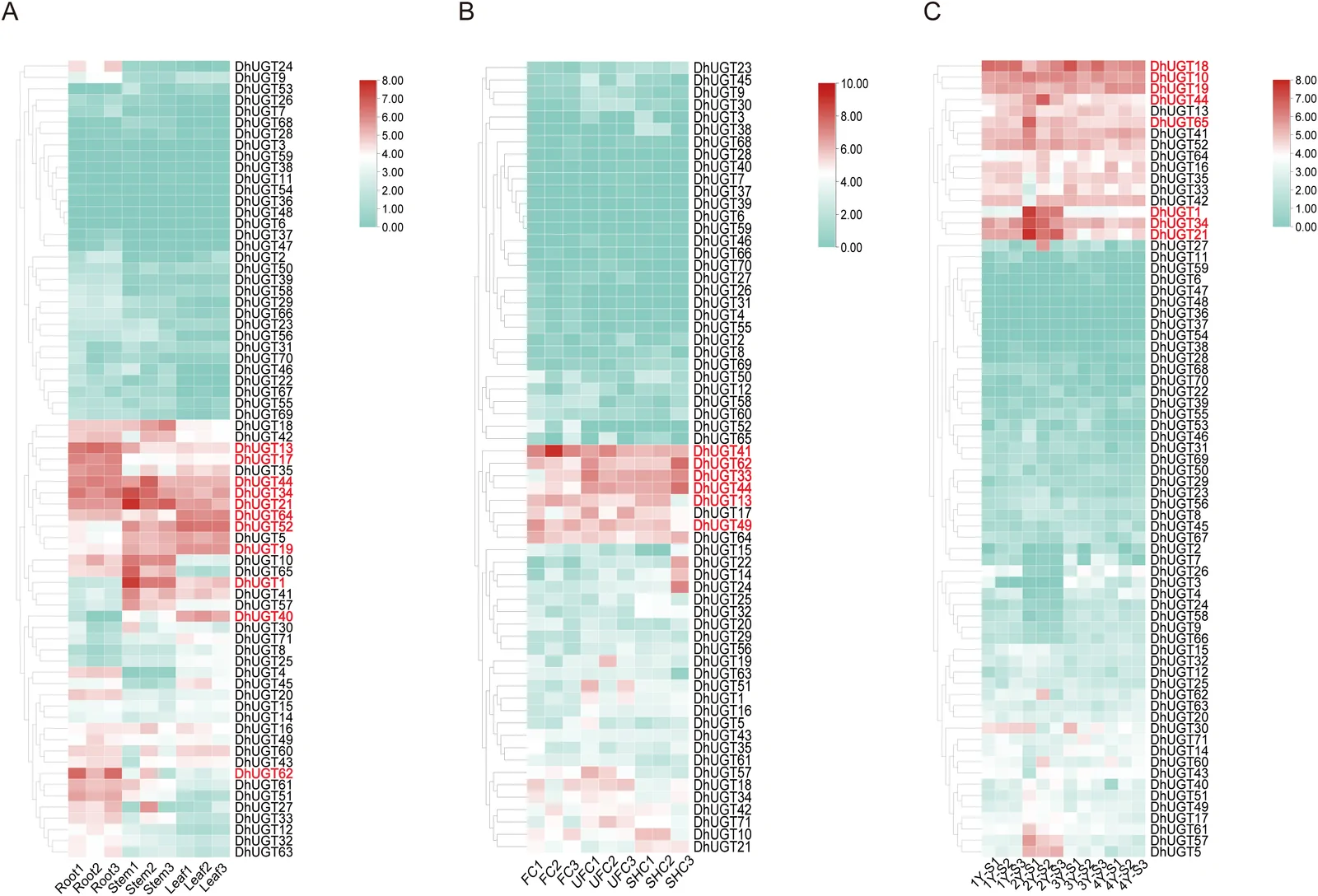
Expression profiles of the *DhUGT* gene family across various tissues, cultivation modes, and developmental stages. **(A)** Heatmap of tissue-specific expression, illustrating the transcript levels of *DhUGT* genes in roots, stems, and leaves. **(B)** Heatmap of expression under different cultivation modes, comparing the transcript variations among simulated habitat cultivation (SHC), facility cultivation (FC), and under-forest cultivation (UFC). **(C)** Heatmap of expression across developmental stages, encompassing stem samples from different growth ages, such as 1-year-old stems (1Y-S) and 2-year-old stems (2Y-S). The color scale of the grids represents expression abundance, with red denoting high expression. All data are based on three independent biological replicates. The underlying expression data used to generate these heatmaps are provided in the **Supplementary Table 7**.

Cultivation modes exerted a profound regulatory effect on the expression profiles of *DhUGT* genes (**Figure 4B**). *DhUGT62* exhibited its highest expression level under simulated habitat cultivation conditions. The expression of *DhUGT13* and *DhUGT49* was significantly induced and upregulated by facility cultivation. *DhUGT33* showed a high expression trend under both under-forest and facility cultivation modes. Notably, *DhUGT41* remained stable and highly expressed across all three cultivation environments. During different developmental stages, the *DhUGT* genes exhibited dynamic expression characteristics correlated with plant age (**Figure 4C**). *DhUGT18*, *DhUGT19*, and *DhUGT10* continuously maintained elevated expression levels throughout the entire 1- to 4-year developmental cycle. The expression levels of *DhUGT44*, *DhUGT65*, and *DhUGT1* peaked in 2-year-old plants and gradually declined thereafter with increasing age. *DhUGT21* and *DhUGT34* retained relatively high transcript levels during the early growth stages (1- to 2-year-old) but were significantly downregulated upon entering the 3- to 4-year-old phase.

By integrating transcriptomic results, seven *DhUGT* candidate genes with robust and stable expression profiles were screened, namely *DhUGT10*, *DhUGT18*, *DhUGT19*, *DhUGT21*, *DhUGT34*, *DhUGT41* and *DhUGT44*. Among these candidates, *DhUGT44* consistently maintained the highest transcript abundance across multiple tissues, diverse cultivation conditions and different developmental stages. Cis-element analysis of the promoter sequences of all seven high-expression candidates confirmed that *DhUGT44* harbored the greatest number of low-temperature responsive elements (LTREs, n = 9), supporting its putative role in the transcriptional regulation of cold stress responses. On the basis of its superior expression stability and marked cold-regulatory potential, *DhUGT44* was selected as the primary candidate for subsequent functional studies.

### 3.7 *In vitro* functional identification of DhUGT44

Recombinant DhUGT44 protein was expressed and purified to homogeneity, with its purity verified by SDS-PAGE (**Figure 5A**). To characterize the catalytic function of DhUGT44, preliminary substrate screening was carried out. Low conversion was detected for naringenin and daidzein, whereas relatively efficient conversion was observed for flavone-type substrates (HPLC chromatograms are shown in **Supplementary Figure 5**, and semi-quantitative conversion rates are summarized in **Supplementary Table 8**). Accordingly, apigenin (1) was used as the sugar acceptor substrate and UDP-Glc as the sugar donor in an *in vitro* enzymatic assay; the schematic diagram of the catalytic reaction and the chemical structure of the product are shown in **Figure 5B**.

**Figure 5:**
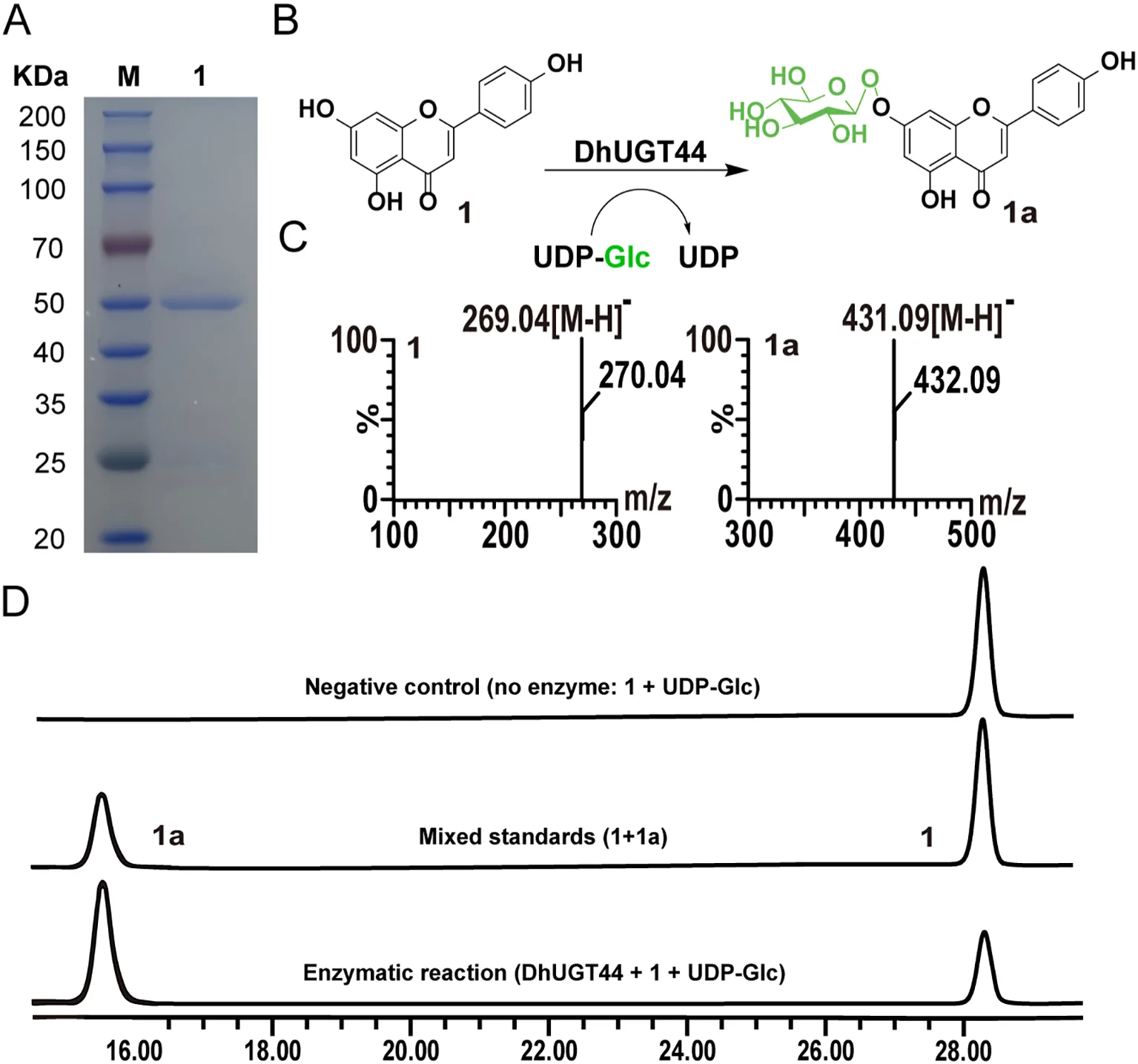
*In vitro* enzymatic analysis of DhUGT44. **(A)** SDS-PAGE analysis of purified tag-free recombinant DhUGT44 protein. **(B)** Schematic diagram of the enzymatic reaction catalyzed by DhUGT44 using apigenin as the sugar acceptor and UDP-glucose as the sugar donor, together with the chemical structure of the product. **(C)** LC–MS spectrum of the enzymatic product acquired in the negative ion mode. **(D)** HPLC chromatogram of enzymatic reaction products.

HPLC analysis revealed a distinct new product peak in the reaction mixture. By comparing retention times with the authentic cosmosiin (apigenin 7-*O*-glucoside) standard, combined with LC-MS analysis, the product was preliminarily identified based on the deprotonated molecular ion at m/z 431.09 [M-H]^-^ in negative-ion mode. Further structural confirmation was achieved by ¹H- and ¹³C-NMR spectroscopy. Detailed chemical-shift assignments for apigenin 7-O-*β*-D-glucopyranoside are summarized in **Supplementary Table 9**, and full raw NMR spectra are provided in **Supplementary Figures 6**, **7**. Collectively, these results demonstrate that DhUGT44 catalyzes the glucosylation of apigenin at the 7-*O*-hydroxyl group (**Figures 5C, D**).

To characterize the enzymatic properties of recombinant DhUGT44, we systematically assessed the effects of pH, reaction time, temperature and metal ions on its catalytic activity using apigenin (1) as the glycosyl acceptor and UDP-Glc as the sugar donor. Full datasets are provided in **Supplementary Figure 9**. The pH profile showed that DhUGT44 achieved maximum activity in Tris-HCl buffer at pH 7.0, indicating that a neutral reaction environment is conducive to its catalytic function. The time-course analysis revealed that the catalytic reaction gradually reached a plateau at 16 h, with no further substantial increase in substrate conversion thereafter. Temperature gradient assays demonstrated that the optimal reaction temperature for DhUGT44 was 40 °C, and its enzymatic activity declined progressively as the temperature rose above 40 °C. With respect to metal ion effects, Ca^2+^ was found to enhance the *in vitro* catalytic activity of DhUGT44.

### 3.8 Subcellular localization and validation of *in vivo* catalytic activity

To elucidate the subcellular site of action of DhUGT44 and validate it’s *in vivo* catalytic function, subcellular localization analysis and transient expression assays were conducted in *N. benthamiana* (**Figures 6A–D**). DhUGT44 was fused with the GFP and transiently expressed in *N. benthamiana* leaves. Laser scanning confocal microscopy (LSCM) observations revealed that the green fluorescent signals of the DhUGT44-GFP fusion protein perfectly co-localized with chloroplasts, overlapping precisely with chlorophyll autofluorescence. Concurrently, a continuous linear fluorescent signal was observed along the cell periphery, suggesting the potential localization of the protein at the plasma membrane.

**Figure 6:**
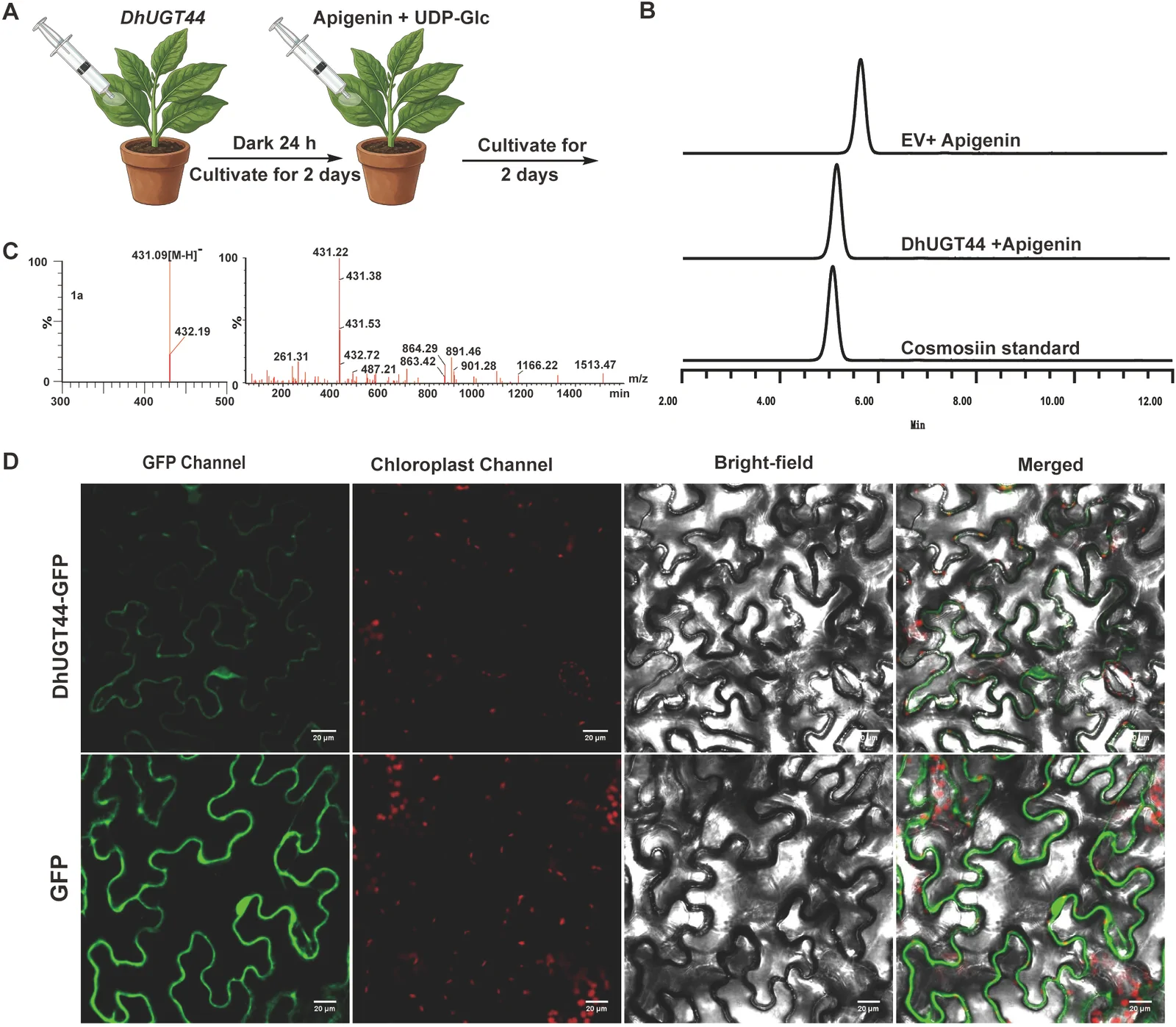
*In vivo* functional verification and subcellular localization analysis of DhUGT44 in *N. benthamiana*. **(A)** Schematic diagram of transient expression and substrate-feeding assays in *N. benthamiana* leaves. **(B)** HPLC chromatograms of the catalytic products produced in *N. benthamiana*. **(C)** LC-MS spectra of the catalytic product accumulated in *N. benthamiana*, acquired in negative ion mode. **(D)** Subcellular localization of DhUGT44 in *N. benthamiana* leaf epidermal cells. GFP, green fluorescent protein; Chloroplast channel, chloroplast autofluorescence; Bright, bright-field image; Merged, merged image of the three channels. GFP driven by the CaMV 35S promoter was used as the control. Scale bar = 20 μm.

To verify the enzymatic activity of DhUGT44 in planta, the substrate apigenin together with the sugar donor UDP-Glc were co-infiltrated into *N. benthamiana* leaves transiently expressing DhUGT44. Following incubation, leaf metabolites were extracted and analyzed using HPLC and LC-MS. HPLC analysis revealed metabolite profiles, while LC-MS was adopted to identify the product and determine its glycosylation site. A distinct chromatographic peak matching the retention time of cosmosiin standard was detected in leaf samples expressing DhUGT44 supplemented with apigenin. In contrast, this peak was absent in empty vector (EV) control leaves treated with apigenin. Consistent with the HPLC results, LC-MS analysis produced a dominant ion signal at m/z 431.09 [M-H]^-^, consistent with the molecular mass of cosmosiin. This mass spectrometric evidence corroborates that the generated product is cosmosiin. Collectively, these data confirm that DhUGT44 efficiently catalyzes the 7-*O*-glucosylation of apigenin (1) to produce cosmosiin *in vivo*, indicating stable and specific glucosyltransferase activity of DhUGT44 both *in vitro* and *in vivo*.

### 3.9 Heterologous expression of *DhUGT44* confers enhanced cold tolerance in *A. thaliana*

To clarify the expression characteristics of *DhUGT44* in response to cold stress, its transcriptional profiles in *D. huoshanense* leaves under cold treatment were examined using quantitative RT-qPCR. The results demonstrated that the transcript abundance of *DhUGT44* changed dynamically over the duration of the cold treatment, peaking at 12 h with an increase of more than 8-fold compared to the 0 h control group. This sharp induction indicates that *DhUGT44* responds sensitively to cold stress signals and actively participates in the low-temperature stress response at the transcriptional level (**Figure 7A**).

**Figure 7:**
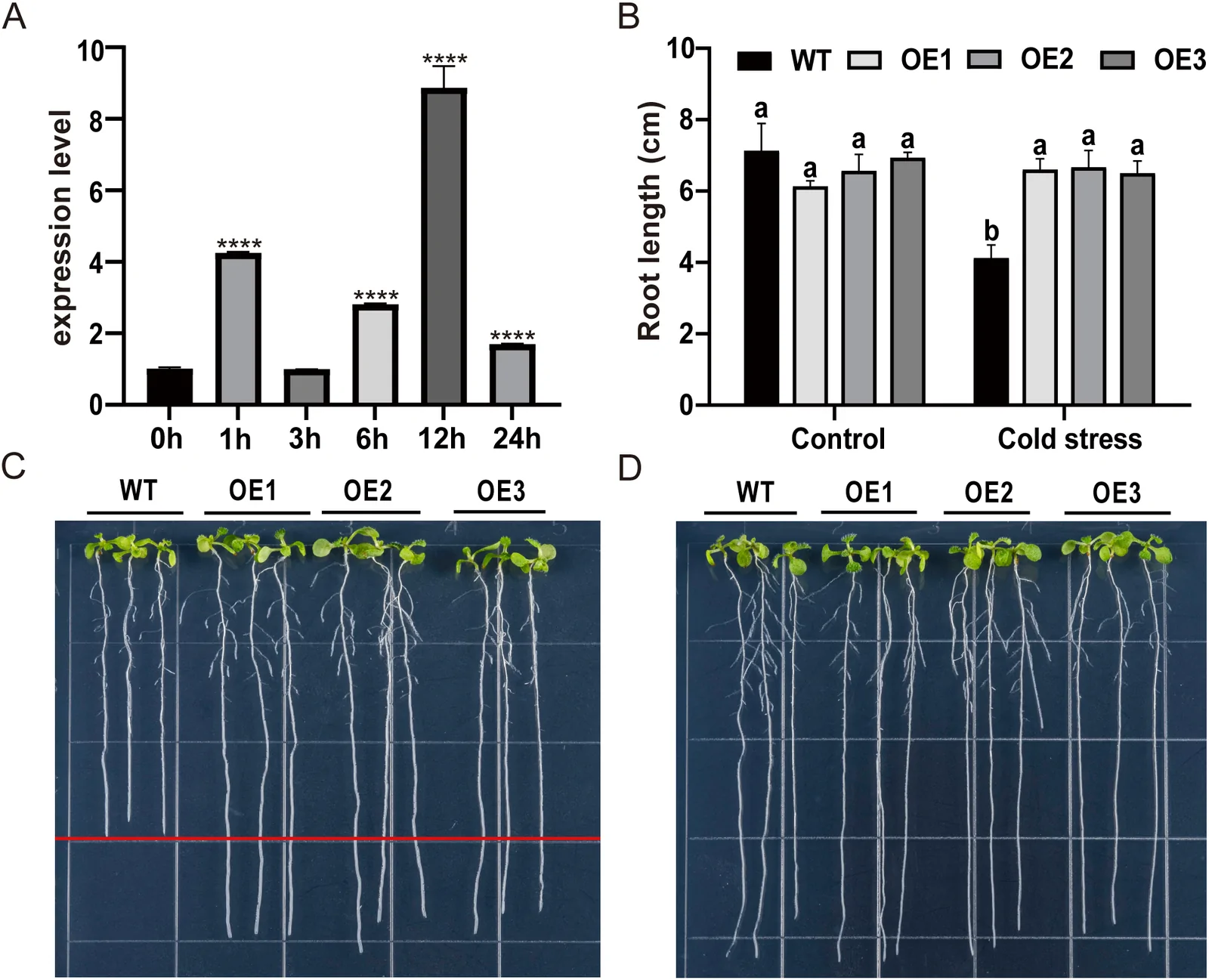
Cold stress response of *DhUGT44* in *D. huoshanense* and functional validation of its role in cold tolerance in transgenic *A. thaliana*. **(A)** Temporal expression profiles of *DhUGT44* in *D. huoshanense* leaves under cold stress. The relative expression levels of *DhUGT44* in leaves were determined via RT-qPCR at 0, 1, 3, 6, 12 and 24 h after cold treatment (4 °C), with the 0 h untreated group serving as the control. The expression data were normalized against the *TBP* gene as the internal reference. Bars represent the mean ± standard error (SE) of three independent biological replicates (n = 3). Different asterisks above the bars indicate statistically significant differences (*P* < 0.0001). **(B)** Statistical analysis of primary root lengths of DhUGT44-OE and wild-type (WT) plants under different treatments. Within each treatment group, different lowercase letters indicate significant differences at *P* < 0.05 according to one-way ANOVA followed by Tukey’s multiple comparison test. **(C)** Root growth phenotypes under cold stress conditions. **(D)** Root growth phenotypes under normal control conditions.

Given this strong transcriptional induction, we subsequently conducted phenotypic observations on wild-type *A. thaliana* and *DhUGT44*-OE lines via vertical 1/2 MS plate culture, to evaluate the heterologous regulatory function of *DhUGT44* under cold stress. Statistical analysis revealed that under cold stress, the average primary root length of the transgenic lines reached 6.50 cm, significantly greater than that of the WT (4.13 cm; *P* < 0.05), functionally validating the positive regulatory role of *DhUGT44* in cold stress tolerance (**Figure 7B**). Consistent with the quantitative results, *DhUGT44*-OE lines exhibited a distinct growth advantage under cold stress, with significantly longer primary roots than WT plants (**Figure 7C**). In contrast, no significant difference in primary root length was detected among all lines under normal growth conditions (**Figure 7D**).

## 4 Discussion

*D. huoshanense*, a rare and endangered medicinal orchid species endemic to China, possesses two key agronomic and medicinal traits: stress tolerance and the biosynthesis and accumulation of characteristic flavonoid glycosides, both of which determine medicinal yield and quality. However, the *UGT* gene family, which mediates the glycosylation of secondary metabolites, has not yet been systematically characterized at the genome-wide level, and functional validation of its members remains limited. This knowledge gap has hindered further studies on stress-resistance breeding and metabolic engineering of bioactive compounds in *D. huoshanense.*

Using the reference genome of *D. huoshanense*, we systematically characterized the evolutionary patterns and molecular features of the *DhUGT* gene family at the genome-wide level through integrated bioinformatic analyses. Focusing on the key family member *DhUGT44*, we further defined its enzymatic function in catalyzing apigenin 7-*O*-glucosylation and verified its positive regulatory role in plant cold tolerance. This systematic investigation, spanning from genome-wide gene family profiling to targeted single-gene functional validation, provides a robust foundation for further dissecting the mechanisms underlying UGT-mediated stress tolerance and quality formation in *D. huoshanense*.

### 4.1 Evolutionary expansion and structural conservation of the *DhUGT* gene family

In this study, a total of 71 intact DhUGT family members were identified within the *D. huoshanense* genome. All identified members harbor the signature PSPG conserved domain characteristic of the UGT family. Phylogenetic analysis revealed that the UGT family in *D. huoshanense* is classified into 12 subgroups and one OG clade. No UGT members were identified in subgroup H, which is presumably caused by gene loss during the evolutionary history of the species. The OG clade, comprising *DhUGT42* and *DhUGT43*, exhibits distinct evolutionary divergence. This phylogenetic branch clusters with *A. thaliana* sterol glycosyltransferase *AtUGT80A2*, suggesting a putative role in sterol glycoside biosynthesis and membrane lipid homeostasis. Subfamily A (*DhUGT16*–*DhUGT19*) is clustered with *AtUGT91A1*, indicating its potential involvement in the glycosylation of phenylpropanoid secondary metabolites.

The vast majority of these genes cluster into the classical A-L subfamilies of angiosperms, suggesting that the core subfamily architecture of the UGT family was established prior to the monocot-dicot divergence (Caputi et al., 2012), and its fundamental functional framework has maintained high functional conservation throughout long-term evolution. Compared to dicotyledonous model species such as *A. thaliana* and *Citrus sinensis*, the *DhUGT* gene family exhibits a relatively compact size, with an expansion pattern distinct from that of dicots. While segmental duplication serves as the primary driver for UGT family expansion in many dicots, the expansion of the DhUGT family is predominantly driven by tandem duplication (Wilson and Tian, 2019), with only a limited number of segmental duplication gene pairs identified. This evolutionary pattern aligns with observations in other monocots, such as rice (*Oryza sativa*) and maize (*Zea mays*), indicating that divergent gene duplication mechanisms have shaped the evolution of *UGT* gene families in monocot and dicot lineages (Li et al., 2014). Previous studies in the congeneric species *Dendrobium officinale* support the premise that tandem duplication is the core mechanism underlying UGT family expansion within the genus *Dendrobium*. These duplication events occurred subsequent to the whole-genome duplication (WGD) event shared by ancestral Orchidaceae, representing a species-specific family expansion during speciation (Niu et al., 2021). Compared with WGD or large-scale segmental duplications, tandem duplication facilitates rapid generation of homologous copies and subsequent functional divergence. This process likely assisted *D. huoshanense* in diversifying its secondary metabolism during its long-term epiphytic evolution, meeting the ecological demands of stress defense (such as low temperature and drought) and the biosynthesis and accumulation of active glycosides-representing a critical evolutionary strategy for adapting to its specialized epiphytic habitat (Zhang et al., 2017).

Gene structure analysis revealed that exons and intron distribution patterns are highly consistent within the same subfamily, with only a few members displaying specific structural variations. This demonstrates that the gene structures of the DhUGT family are highly conserved and streamlined overall. In Orchidaceae, stress-responsive genes typically feature a compact, intron-poor structure, which likely underpins their ability to mount rapid transcriptional responses to drought, low temperature, salicylic acid, and other environmental stimuli. This genomic streamlinedness reduces the temporal cost of gene expression, allowing efficient activation of secondary metabolism and stress defense pathways—an adaptive feature consistent with the epiphytic lifestyle of *D. huoshanense*. Among the conserved motifs, Motif 1-functioning as the core catalytic domain-is highly conserved across all family members, thereby ensuring that each DhUGT possesses fundamental glycosyltransferase activity. In contrast, the subfamily-specific motifs may serve as the primary molecular basis underlying the substrate preference and functional divergence observed among different DhUGTs.

### 4.2 Cis-acting element and transcription factor analysis reveals multiple regulatory modes of *DhUGTs*

Systematic analysis of cis-acting regulatory elements (CREs) and TFBS is a cornerstone for functional prediction of genes and the structure of transcriptional regulatory networks. Our analysis revealed that the promoter regions of the 71 *DhUGT* genes universally contain regulatory elements responsive to light, phytohormones, and abiotic stresses. Concurrently, distinct variations in element composition among different subfamilies suggest a highly diversified transcriptional regulatory mode within this gene family. This transcriptional diversity provides a molecular foundation for their broad involvement in various biological processes, including secondary metabolic modification, growth and development, and stress adaptation. Notably, light-responsive elements are ubiquitously present across all DhUGT promoters, representing the most widely distributed element type in this family. As a typical shade-demanding epiphytic plant, *D. huoshanense* is adapted to forest understory low-light environments, where light signals serve as critical environmental cues dictating plant growth, development, and the accumulation of secondary metabolites. Previous studies in model and medicinal plants, such as *A. thaliana* and *Salvia miltiorrhiza*, have confirmed that light signals directly modulate the rate of biosynthesis and accumulation of active flavonoids and glycosides by regulating the transcription levels of specific UGTs (Stracke et al., 2010). Consequently, the widely distributed light-responsive elements in the DhUGT promoters likely serve as the core regulatory platform for responding to light fluctuations and modulating active glycoside biosynthesis, thereby ensuring the uninterrupted operation of secondary metabolic pathways in its native low light habitats.

DhUGT promoter regions exhibit pronounced enrichment of hormone-responsive cis-elements for ethylene, MeJA and ABA, as well as cognate transcription factor binding sites including ERF, WRKY and bZIP. Combined, these components form a sophisticated regulatory framework controlling DhUGT activity under abiotic stress (Chen et al., 2023b). *D. huoshanense* naturally epiphytitizes on tree trunks or rock surfaces where it lacks the moisture-buffering capacity of soil, leaving it highly vulnerable to adverse environmental stresses such as low temperature and drought. Phytohormone signaling represents the primary channel mediating plant stress responses. In these pathways, *UGT* genes act as downstream functional targets, participating in drought and cold stress adaptation via ERF-mediated ethylene signaling pathways. Within the DhUGT family, ERF binding sites exhibit a significant co-enrichment pattern with ethylene-responsive elements, suggesting that members of this family are directly regulated by ERF-type transcription factors to participate in ethylene-mediated stress response cascades.

In addition, binding sites for the BBR-BPC family occurred with the highest frequency across the DhUGT family. The BBR-BPC family is not only involved in the basal regulation of plant growth and development, but also functions as key signal integrators that coordinately modulate hormone signal transduction and secondary metabolic pathways. Furthermore, binding sites for secondary metabolic regulators, such as MYB and bHLH, are widely distributed and structurally aligned with light- and stress-responsive elements (Yu et al., 2025). Empirical evidence from *Juglans regia* (Zhang et al., 2025) and *Salvia miltiorrhiza* (Shi et al., 2022) has confirmed that MYB transcription factors directly bind to UGT promoters to positively regulate the accumulation of flavonoid glycosides. Taken together, these findings strongly suggest that the BBR-BPC family acts as an upstream master regulator governing DhUGT expression, and that the MYB-UGT regulatory module plays an indispensable role in the glycosylation of active flavonoids in *D. huoshanense.* We propose that this family coordinates the expression of multiple *DhUGT* genes, thereby integrating secondary metabolism and stress adaptation responses in this epiphytic orchid.

### 4.3 Molecular mechanism speculate and functional characterization of *DhUGT44* in response to cold stress

Cis-acting element analysis revealed that the promoter regions of the *DhUGT* gene family are highly enriched with various abiotic stress-responsive motifs, strongly indicating that this family exerts a broad regulatory role in environmental adaptation. As core functional genes in plant stress responses, members of the multi-functional UGT family have been extensively demonstrated to mitigate diverse stress crises by mediating the glycosylation of secondary metabolites. For instance, overexpression of rice *UGT706F1* (Zhao et al., 2025b) and *Carex rigescens CrUGT87A*1 (Zhang et al., 2021) significantly enhan*ces plant* tolerance to heat and salt stresses, primarily by driving the accumulation of flavonoids and boosting antioxidant capacity. Similarly, wild barley *HvUGT1* (Feng et al., 2024) and rice *UGT85E1* (Liu et al., 2021) are c*apable* of mainta*ining c*ellular homeostasis under drought stress via the activation of reactive oxygen species (ROS) scavenging systems. Collectively, these findings substantiate that UGT-mediated glycosylation of metabolites represents a highly conserved and efficient regulatory pathway for plants to cope with abiotic stresses.

As a critical environmental factor limiting the growth and quality of *Dendrobium* species, low-temperature stress typically triggers the excessive accumulation of intracellular ROS, thereby disrupting cell membrane integrity and cellular homeostasis (Sachdev et al., 2021). To date, the metabolic and physiological responses of various *Dendrobium* species to cold stress have been extensively documented. For instance, upon exposure to 4°C cold treatment, *Dendrobium officinale* harboring genes such as *DcPP2C5* and *DoCDPK1* can maintain cellular homeostasis and osmotic balance by enhancing the activities of antioxidant enzymes (e.g., SOD, POD, and CAT) while simultaneously enriching soluble sugars, proline, and flavonoids (Bao et al., 2024). In *Dendrobium nobile*, cold stress significantly induces key transcription factors and genes involved in starch and sucrose metabolism pathways. These genes include loci such as *TPP6* and *DPE1*, which thereby drive the substantial accumulation of polysaccharides, flavonoids, and polyphenols (Fan et al., 2024). Furthermore, previous omics studies on *D. huoshanense* revealed that 4°C cold stress markedly activates flavonoid biosynthesis and plant hormone signal transduction pathways, leading to elevated contents of mannose and total flavonoids (Wu et al., 2023). Collectively, these findings indicate that the modification and accumulation of flavonoids and polysaccharides may represent a core, shared strategy for *Dendrobium* species to cope with low-temperature adversity.

Based on this theoretical background, the present study successfully identified *DhUGT44* as a crucial cold responsive candidate gene through a combination of genome wide identification and expression profiling analyses. RT-qPCR validation confirmed that *DhUGT44* exhibits a typical early stress responsive pattern, with its expression level being strongly induced by low temperature. The DhUGT44 protein is dually localized to the plasma membrane and chloroplasts. The plasma membrane serves as a key structure for plant perception and response to low temperature signals, while chloroplasts are the main organelles where cold-induced ROS burst occurs, and oxidative damage is an important physiological mechanism underlying growth inhibition under cold stress. Combined with the verified 7-*O*-glycosyltransferase activity of DhUGT44 toward flavonoids, we hypothesize that the increased expression of *DhUGT44* under low temperature stress enables its encoded enzyme to specifically catalyze 7-*O*-glycosylation modification of flavonoid aglycones. On the one hand, such modification markedly enhances the water solubility of flavonoid aglycones and facilitates their subcellular trafficking, allowing efficient accumulation of these compounds at key sites of ROS burst such as the plasma membrane and chloroplasts to exert *in situ* antioxidant effects. On the other hand, glycosylation stabilizes the molecular structure of flavonoids and prolongs their intracellular half-life, thereby continuously maintaining cellular redox homeostasis and plasma membrane integrity. Collectively, these biochemical effects alleviate low temperature triggered oxidative damage and growth inhibition, and ultimately positively regulate plant cold tolerance. This also provides a theoretical interpretation for the phenotypic observations obtained from subsequent heterologous overexpression.

Although our study has preliminary established that *DhUGT44* possesses both flavonoid 7-*O*-glycosyltransferase activity and cold-resistance functions, the complete molecular regulatory network governing this gene remains to be fully elucidated, revealing certain limitations in the current work. First, the upstream transcriptional regulatory mechanism remains limited to bioinformatic predictions; the specific binding relationships and transcriptional activation effects between the cold-responsive elements enriched in the *DhUGT44* promoter and potential interacting transcription factors have not yet been validated via experimental systems such as yeast one-hybrid (Y1H) or dual-luciferase reporter assays, leaving the core transcriptional module mediating cold-induced *DhUGT44* expression unverified. Second, we still lack sufficient evidence for downstream metabolic regulation. We have only characterized the enzyme’s catalytic activity on its substrates *in vitro*, have not quantified key physiological and metabolic parameters in cold-stressed transgenic plants, including endogenous flavonoid glycoside contents, *in vivo* ROS scavenging efficiency, and antioxidant enzyme activities. To address these current limitations, subsequent work can leverage a stable genetic transformation system for *D. huoshanense* to resolve the glycosylation preferences of *DhUGT44* toward flavonoid and polysaccharide substrates in homologous native plants. Concurrently, by integrating multi-layered technical approaches including targeted metabolomics and multi-stress transcriptomics, future research will comprehensively dissect the upstream and downstream molecular pathways of *DhUGT44*, thereby systematically elucidating its biological functions.

## 5 Conclusions

In this study, the *UGT* gene family of the medicinal plant *D. huoshanense* was systematically identified at the genome-wide level for the first time, resulting in the identification of 71 *DhUGT* members. Through phylogenetic analysis, conserved motif characterization, and multidimensional transcriptomic profiling across various tissues, cultivation modes, and developmental stages, *DhUGT44*, a member of phylogenetic group L, was targeted as the core candidate gene involved in the low-temperature response. Functional characterization revealed that DhUGT44 localizes to the plasma membrane and chloroplasts and exhibits flavonoid 7-*O*-glycosyltransferase activity. Heterologous overexpression of *DhUGT44* in *Arabidopsis* significantly enhanced root growth and cold tolerance under low-temperature stress. Collectively, this study not only expands our understanding of the evolution and function of the *UGT* gene family in Orchidaceae medicinal plants, but also provides key genetic resources and a theoretical foundation for the genetic improvement of stress tolerance and metabolic engineering of active flavonoids in *D. huoshanense*.

## Data Availability

The datasets presented in this study can be found in online repositories. The names of the repository/repositories and accession number(s) can be found below: NGDC, CRA000551 and CRA005817, and NCBI PRJNA1189807.

## Supplementary material

Supplementary Figure 1 Hygromycin resistance screening of transgenic *Arabidopsis thaliana* seedlings. Resistant transgenic seedlings developed normal green cotyledons and root systems. This image is representative of three independent biological replicates with consistent results

Supplementary Figure 2 Analysis of conserved motifs in *DhUGT* protein sequences.

Supplementary Figure 3 Distribution of cis-acting elements in the 2-kb upstream promoter regions of *DhUGT* genes. Different colors represent distinct responsive elements.

Supplementary Figure 4 Prediction of transcription factor binding sites within the 2-kb upstream regulatory regions of *DhUGT* genes. Different colored boxes represent distinct transcription factor binding sites.

Supplementary Figure 5 HPLC chromatograms of glycosylation reactions catalyzed by DhUGT44. **(A)** Naringenin reaction system; **(B)** Daidzein reaction system; **(C)** Norwogonin reaction system.Each panel: upper trace, enzymatic reaction mixture.

Supplementary Figure 6 ^13^C‑NMR spectrum of apigenin 7‑O‑glucoside (125 MHz, DMSO‑d_6_).

Supplementary Figure 7 ^1^H‑NMR spectrum of apigenin 7‑O‑glucoside (500 MHz, DMSO‑d_6_).

Supplementary Figure 8 Enzymatic properties of recombinant *DhUGT*. **(A)** Reaction time course; **(B)** pH and buffer system effects; **(C)** metal ion and EDTA effects; **(D)** temperature effects. Data are shown as mean ± SD (n = 3), with the highest activity set as 100%.
